# Influence of Dynamic Balance on Jumping-Based Asymmetries in Team Sport: A between-Sports Comparison in Basketball and Handball Athletes

**DOI:** 10.3390/ijerph18041866

**Published:** 2021-02-14

**Authors:** Francisco J. Barrera-Domínguez, Antonio Carmona-Gómez, Inmaculada Tornero-Quiñones, Jesús Sáez-Padilla, Ángela Sierra-Robles, Jorge Molina-López

**Affiliations:** 1 Faculty of Education, Psychology and Sport Sciences, University of Huelva, 21071 Huelva, Spain; fbarreradominguez@gmail.com (F.J.B.-D.); antoniocarmonagomez@gmail.com (A.C.-G.); inmaculada.tornero@dempc.uhu.es (I.T.-Q.); jesus.saez@dempc.uhu.es (J.S.-P.); sierras@dempc.uhu.es (Á.S.-R.); 2 Institute of Nutrition and Food Technology, Biomedical Research Centre, Health Sciences Technological Park, University of Granada, 18010 Granada, Spain

**Keywords:** inter-limb asymmetry, functional test, sports performance, lower limb strength

## Abstract

The aims of the present study were to analyze mobility, dynamic balance and lower-limb strength and the prevalence of asymmetry according to the type of sport and assess the association between inter-limb asymmetry and sports performance. A total of 23 basketball and 25 handball players performed a test battery consisting of functional movements and a jump test. Inter-limb asymmetry was calculated using a standard percentage difference equation. A between-groups comparison analysis was carried out, and Pearson’s correlation coefficients (r) were calculated to establish a relationship between asymmetries and physical performance. The results found athletes in different sports to exhibit different performance in functional movements and the jump test, but no bilateral asymmetries. The reactive strength index (RSI) and stiffness asymmetries were significantly associated with the anterior reach Y-balance test (YBT) (r = −0.412; *p* < 0.01 and r = −0.359; *p* < 0.05, respectively), and the unilateral triple hop test (THTU) was negatively correlated to anterior reach, posterior lateral reach YBT and YBT composite YBT (r = −0.341 to −0.377; *p* < 0.05). These findings suggest that the asymmetries exhibited important dispersion not dependent upon the type of sport but on each individual and the applied test. In addition, asymmetry in anterior direction YBT showed the strongest association to the rest of the sports performance variables.

## 1. Introduction

Team sports are characterized by high-intensity unilateral actions such as sprints, jumps or changes of direction (COD) [1]. On-court sports such as basketball and handball are two different team sports; however, given their characteristics and rules, many game actions, physiological demands and movement patterns are similar [2,3]. Speed actions, lateral movements, jumps or COD are skills common to both modalities and determinants of their athletic demands [2]. Thus, athletes may require adequate neuromuscular coordination and motor control to produce high levels of specific unilateral strength during game actions [4]. Recent research shows that these actions are unlikely to occur in a similar amount for both extremities [5,6]. Therefore, it has been suggested that unilateral actions develop and increase inter-limb imbalances in team sports, creating a dominant side [7]. The existence of inter-limb asymmetries, therefore, would be expected in team sports athletes [8].

Inter-limb differences and their relation to sports performance have been widely addressed in the scientific literature [9]. On one hand, a high imbalance between legs in relation to sport performance variables such as strength or speed has been marginally associated with a decrease in sports performance [7,10]. On the other hand, inter-limb asymmetries in strength or power have also been proposed as a major risk factor for sports injuries [11]. Specifically, the literature has described various thresholds that have been widely evaluated, establishing that athletes with over 10% [12] or 15% [11] of inter-limb asymmetries may have a higher incidence of injuries compared to those below these thresholds. Therefore, an increase in inter-limb symmetry could be considered as a marker of success in the re-adaptation process since it enhances the athlete’s confidence and makes a return to play safer and more effective [11]. In a critical review, Maloney [13] pointed out that sports professionals should consider that the weakest limb presents a greater “window of opportunity” with which to improve overall performance. However, when the asymmetry between legs is analyzed and compared using different tests, the results prove inconsistent, and the dominant leg may even change depending on the test used [14]—thus suggesting a clear test-dependent relationship regarding the presence of asymmetry. Despite the prior evidence on inter-limb asymmetry in a wide range of team sports [14,15], few studies have analyzed asymmetries in handball and established comparisons with a similar on-court sport such as basketball.

Given the usefulness of clarifying the role of inter-limb asymmetry in sports performance for coaches and trainers, interest in the topic has grown. In reference to the factors affecting athletic performance, previous evidence has shown that functional tests such as dorsiflexion or dynamic balance asymmetries could be related to an increase in strength values during sprinting [16], as well as to decline in sports performance related to COD actions or to vertical jump through the countermovement jump (CMJ) [17]. With respect to asymmetry measured through jumping actions in both force vectors, several authors have reviewed the existing evidence, with contradictory results [11,13].

Based on the above, the existing evidence yields inconclusive results when trying to determine the association between limb asymmetry and sports performance measures and indicates that the observation of asymmetry alone offers little information for sports physicians [8]. In addition, the important heterogeneity observed in terms of leg asymmetry, even within the same team, could make it difficult to establish relationships between inter-limb asymmetry and its correlation to usual performance parameters. The observed contradictions may be due to age, gender, the tests used to evaluate asymmetries, or the sport-specific characteristics of the sample analyzed. Moreover, there is a dearth of studies investigating the relationship between limb asymmetries and performance tests in handball athletes. Therefore, the present study was designed to (i) determine mobility, dynamic balance and lower-limb strength and the prevalence of asymmetry according to the type of sport; and (ii) to assess the association between inter-limb asymmetry and different sport performance variables.

## 2. Materials and Methods

### 2.1. Design

A cross-sectional was employed to determine the mobility, the dynamic balance, the lower-limb strength and the prevalence of asymmetry according to the type of sport, as well as the association between inter-limb asymmetry and different sport performance variables, respectively. All tests were performed in November 2019. The procedures followed the rules defined by the Declaration of Helsinki. This study was authorized by the Andalusian Committee of Ethics in Biomedical Research (reference number: FBD_UHU2020).

### 2.2. Participants

Forty-eight competitive team sports athletes, specifically basketball (n = 23) and handball (n = 25) (mean age: 18.98 ± 3.97 years; mean height: 181.40 ± 7.69 cm; mean body weight: 78.45 ± 13.62 kg) were informed and gave their consent to participate in this study. Subjects were assessed for eligibility if they had at least 5 years of previous experience; played at a federated level; had regular training three days or more per week during the study period. Subjects were excluded if they presented any injury during and at least 6 months prior to the study; they were not in good health or did not pass a medical exam before the season.

### 2.3. Procedure

One week before the data collection, all participants were familiarized with the performance test procedures enabling them to perform each test between 3–6 times. During the testing days, all players completed a previously published standardized warm-up [18] consisting of 5 min low-intensity with the ball, 5 min of hip mobility and core activation, 5 min of low-impact landings, accelerations and plyometrics. Following the warm-up, subjects were allowed to train the trials executions for at least 5 min and providing them the feedback for proper technical execution. Subjects refrained from intensive exercise and stimulants in the 24-hour period prior to testing execution. Testing was divided into two separate days with 24 h–48 h in between. The first session involved the body composition measures, the WB-DF and the YBT tests. The second session involved the jump tests: CMJ, unilateral drop jump (DJU) at the height of 25 cm and unilateral triple hop test (THTU).

### 2.4. Outcomes Assessment

#### 2.4.1. Body Composition Measures

Initially, and prior to body composition, body height was determined with a stadiometer (0.1 cm, Secca 220, Spain). Later, multi-frequency bioelectrical impedance equipment (Inbody 230 Multi-frequency segmental body composition analyzer, Barcelona, Spain) was used to determine the body composition. All players had to remain static in an anatomical position with arms and legs slightly separated and placed on the electrodes. The analyzer complies with the European standards for use in the medical industry (93/42 EEC, 90/384 EEC). Weight, body mass index (BMI), lean mass, and fat mass expressed as a percentage of total body fat were determined for further analysis.

#### 2.4.2. Weight-bearing Dorsiflexion Test (WB-DF)

Dorsiflex iPhone App [19] was used to determine ankle dorsiflexion. Initially, each player was instructed to perform the test with their hands on their hips, putting the assigned leg in front and the opposite leg is placed one foot behind, both legs are placed at the width of the hips. From this position, the athlete had to bring the knee forward without lifting the heel off the ground. The mobile device was placed with the screen touching the tibia under tibial tuberosity, showing the result of ankle dorsiflexion in degrees. All the players performed the test barefoot, three attempts per leg were measured, and the average of the three was calculated for further analysis.

#### 2.4.3. Y-Balance Test (YBT)

Dynamic balance was evaluated by using the OctoBalance device (OctoBalance, Check your Motion, Albacete, Spain) through a modified version of the SEBT [20]. To assess dynamic balance, subjects are asked to push the point of the tape measure with their toes as far as possible in the designated direction (anterior, posteromedial, and posterolateral) while balancing with the other leg on the anteroposterior line of the platform. In order for each attempt to be considered valid, the players had to keep their hands on their hips during the test, do not raise the heel of the platform, and also the foot that reaches cannot be supported off the platform. Before starting, the measuring tape was placed at a distance of 30 cm, which is the minimum distance to be reached. Measurements were made only once per leg, as these were experienced subjects [20]. The laterality of the test was defined by the leg that was supported in the octagon at the moment of the reach.

#### 2.4.4. Triple Hop Test for Distance Unilateral (THTU)

THTU was carried out to evaluate the elastic-reactive force of the lower extremities in the horizontal axis [21]. Before starting, each participant positioned the leg to be measured, resting the foot on the starting line, and each player performed three consecutive maximum forward jumps with the same limb. Then, the researcher determined the total distance from the start to the point where the heel hit the ground upon completion of the third jump with a tape measure [21]. Arms swinging was allowed during the test to balance, but the attempts were considered failed if the participant lost balance during any part of the test or was unable to maintain the final posture for at least two seconds. Each athlete performed the test three times with a break of at least two minutes between each attempt, and finally, the best attempt was selected.

#### 2.4.5. Countermovement Jump Test (CMJ)

Vertical jump height was measure using a Chronojump (Chronojump Bosco-System^®^, Barcelona, Spain) [22]. Within the platform, players executed a jump with a countermovement seeking the greater height. For the jump to being considered valid, it must be done with the hands on the hips, with the knees extended during the flight time, and the player must land at the same point where the jump was made. If these items were not achieved, the attempt was invalid and must be repeated. Each player made a total of three valid attempts, and the best trial was used for further analyses.

#### 2.4.6. Unilateral Drop Jump Test (DJU)

The unilateral jump test was assessed with a Chronojump (Chronojump Bosco-System^®^, Barcelona, Spain) [22]. The initial height of fall was 25 cm, from where subjects were asked to jump as high as possible with each leg. In each attempt, it was indicated that both hands must be placed on the hips, the knees must be extended when the foot not in contact with the platform, and both landings must be made at the same point. A total of 3 jumps were performed with each leg, with approximately 2 minutes of recovery in between. Finally, the best of these attempts was used for a more detailed analysis. In addition to the height of the jump, other variables were extracted, such as the reactive strength index (RSI) that was calculated using the relationship between flight time and contact time, and vertical stiffness that was calculated considering the contact time, time of air and body mass and using the equation of Morin et al. [23].

### 2.5. Statistical Analyses

All statistical analyses were computed using the Statistics Package for Social Sciences version 25 statistical package (SPSS, IBM, Chicago, IL, USA). Descriptive statistics were derived (Mean, standard deviation, the 95% confidence limit (CL) and the range), and due to sample size, a Shapiro–Wilk test was used to check the data distribution of the tested parameters. A between-groups comparison analysis was performed to explore the basketball versus handball differences in mobility, dynamic balance, lower limb strength and asymmetries. The model was adjusted by age. Moreover, the magnitude of the difference in the analyzed parameters was determined using Hedge’s effect sizes (ES) on the pooled SD together, and the 90% CL was calculated for all dependent variables. Probabilities were also calculated to determine whether true (unknown) differences were smaller, similar or larger than the smallest worthwhile difference or change (0.2 x SD between subjects) [24]. Values were interpreted as follows: < 1%, almost certainly not; 1–5%, very unlikely; 5–25%, unlikely; 25–75%, possible; 75–95%, likely; 95–99%, very likely; and > 99%, most likely [25,26]. Finally, if the chances of obtaining a beneficial/better or detrimental/worse were both > 5%, the effect was assessed as unclear [27,28]. Pearson’s correlations (r) were computed to compare scores on asymmetries and their relationship to sports performance parameters. The magnitude of Pearson’s correlation was interpreted as 0.0–0.1 = trivial; 0.1–0.3 = small; 0.3–0.5 = moderate; 0.5–0.7 = large; 0.7–0.9 = very large and 0.9–1.0 = almost perfect [27]. Statistical significance was established at *p* < 0.05.

## 3. Results

The descriptive results referred to sample data, mobility, dynamic balance, and lower limb strength are reported in Table 1.

Table 2 shows the comparative analysis of the mean values referred to mobility, dynamic balance, strength, and their respective asymmetries according to the type of sport. Regarding mobility, no changes were observed for the analyzed variables depending on the type of sport; however, the prevalence of asymmetry was 22.8% for ankle dorsiflexion. Regarding dynamic balance, basketball players showed better scores in the YBT test in all directions as well as in the overall test score. The scores in absolute values showed a change ranging from 6.23 to 14.6 percent for this test. However, a total of 16.5% of the total sample revealed asymmetries for the dynamic balance. Finally, the comparative analysis of lower limb strength revealed differences between sports for most of the parameters studied in the vertical and horizontal jump, with the exception of the CMJ, DJU R and THT L. Specifically, basketball players showed the highest percentage change for stiffness (between 32.1 to 39.5). A total of 65.3% of the athletes exhibited asymmetry of the RSI variable.

**Figure 1:**
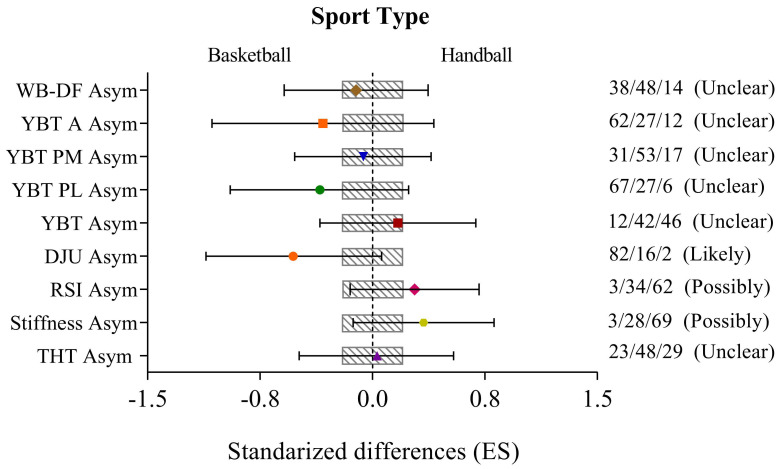
Comparative analysis of mobility, dynamic balance and lower limb strength asymmetries according to the type of sport. The model was adjusted for age. Bars indicate uncertainty in the true mean changes with 90% confidence intervals. Trivial (shaded) areas were calculated from the smallest worthwhile change. Quantitative chances of better or worse effects were determined as follows: <1%—almost certainly not; 1–5%—very unlikely; 5–25%—unlikely; 25–75%—possible; 75–95%—likely; 95–99%—very likely; and >99%—most likely [25,26]. Asym—asymmetry; WB-DF—weight-bearing dorsiflexion; YBT A—Y-balance test anterior reach; YBT PM—Y-balance test posteromedial reach; YBT PL—Y-balance test posterolateral reach; YBT—Y-balance test composite of all directions; DJU—drop jump unilateral; RSI—reactive strength index; THT—triple hop test; ES—effect size.

Figure 1 shows the comparative analysis of asymmetries referred to mobility, dynamic equilibrium and strength in the lower limbs according to the type of sport. The results revealed differences possibly due to the type of sport for DJU-derived parameters such as reactive strength index (31.96% (CL 90%: −13.99; 102.47)) and stiffness (50.31% (CL 90%: −14.47; 164.13)). Furthermore, when the vertical drop jump height was considered to assess asymmetry, the differences observed in the basketball players were probably due to the specificity of this sport (−30.86% (CL 90%: −54.20; 4.38)). Finally, unclear or improbable effects were observed for the rest of the analyzed parameters according to the type of sport.

**Figure 2:**
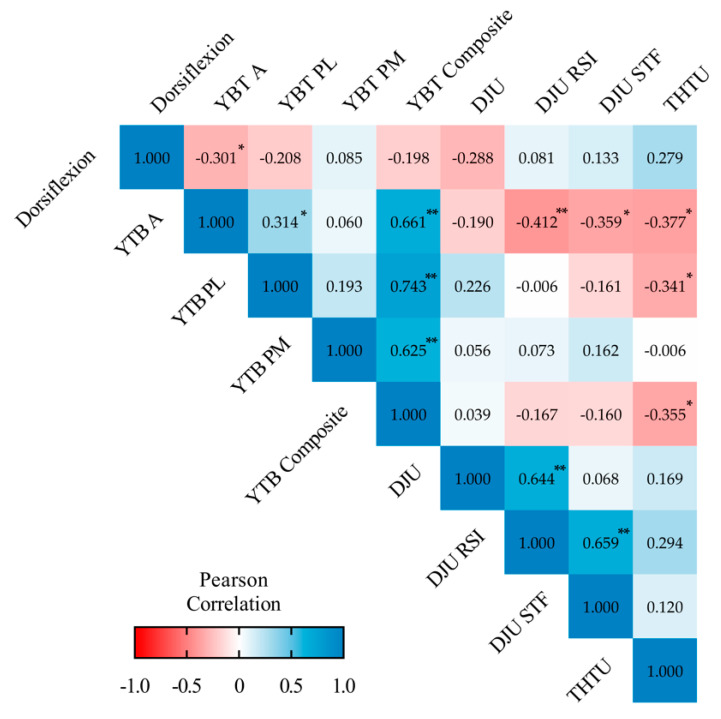
Matrix correlations (Pearson’s r, 95% confidence intervals) between the inter-limb asymmetry scores in functional tests and lower limb strength sports performance. The model was adjusted for age. * Statistical significance at *p* < 0.05. ** Statistical significance at *p* < 0.01. YBT A—Y-balance test anterior reach; YBT PM—Y-balance test posteromedial reach; YBT PL—Y-balance test posterolateral reach; YBT—Y-balance test composite of all directions; DJU—drop jump unilateral; RSI—reactive strength index; STF—stiffness; THTU—triple hop test unilateral.

Figure 2 shows a heat map correlation analysis between functional test and lower limb strength according to vertical and horizontal jump. The asymmetry in dorsiflexion was negatively correlated to scores in the anterior YBT test. In addition, dynamic stability determined by the YBT test was negatively correlated to DJU RSI, DJU STF and THTU in both the anterior and the posteromedial planes, as well as in the composite YBT score. In addition, the asymmetries of the YBT test in the different planes were found to be positively correlated to each other, as well as to the composite YBT. Similarly, positive correlations were observed between de different DJU parameters.

## 4. Discussion

One of the main findings of the study was that the type of sport influences dynamic equilibrium in all the evaluated directions and the DJU measured parameters, where the scores of the basketball players were better than those of the handball counterparts. In contrast, handball players demonstrated greater strength in the horizontal vector as evaluated through the THTU. On the other hand, it also has been observed the relationship between the asymmetries in functional tests and athletic performance variables. A moderate and negative correlation was observed between asymmetries in YBT performance, specifically in the anterior plane, and RSI, stiffness and the THT tests. This suggests that large asymmetries in the anterior direction may be related to a reduction in athlete performance as assessed by RSI, stiffness and THT (in absolute values)—these being variables previously described as important in the performance of team sports such as basketball and handball [3].

Both sports, basketball and handball, are team sports whose intensity pattern has been defined as intermittent (stop and go), and where the game itself demands mainly sprint actions, lateral movements, jumps and COD. Thus, while these are two different sports, their physiological demands and movement patterns are similar [2,3]. Even so, the evaluated dynamic balance and jumping differed on comparing them—probably because of the specificity of each sport. In this respect, our results evidenced higher performance in dynamic balance and vertical jumping actions among the basketball players; whereas, handball players achieved better performance in triple horizontal jump actions. This could be explained by previous research [29,30] that has shown a lower amount of jumping actions in handball than in basketball. Likewise, jumps in handball requires a greater demand for the horizontal component of the force since the rules allow greater contact actions through powerful lower-body movements as well as tackles of opponents, sideways running, backward running, or CODs with a high number of physical confrontations with the opponent players [31]. In contrast, in basketball, the fact that the rules are less permissive with the contact between players and the vertical backboard location will allow a greater demand for the force to be found in the vertical component [32]. These results, therefore, could confirm that the physical characteristics of strength or dynamic balance are highly specific and depend on the type of sport involved, even though comparison according to bilateral asymmetries in the lower limbs showed no differences regarding the type of sport practiced. Considering both our findings and previously published evidence [33] comparing the performance of athletes in 14 different sports referred to as lower extremity strength and dynamic balance, it could be possible to establish sport-based standardized data in terms of absolute values. Nevertheless, the lack of effect of the sport practiced upon bilateral asymmetry in the lower limbs and the range of equations applied to define asymmetries among athletes in different sports [17,34,35,36] make it necessary for the asymmetry values to be analyzed in detail and monitored individually for each athlete [33].

Previous studies have shown asymmetry between legs to be closely associated with an athlete’s risk of injury [11,37,38]. However, given the important variability shown by asymmetry within the same sport and even within the same player [8,11,39,40], it would be premature for coaches and physicians to provide a standardized threshold above which the risk of injury increases. Furthermore, other authors [41,42] indicate that the interlimb differences should be considered as asymmetry only when they are greater than intralimb variability. Thus, it seems that there is no consensus in the scientific literature on a specific threshold to determine asymmetry, although a large part of research often proposes that 10–15% is the limit to defining a player as presenting asymmetry [11,12]. Both our results and those of other authors [33] would confirm that this threshold could differ depending on the morphological demands of the sport itself and even within the same team, given the individual demands of each player on the court [40,42]. In our study, the analysis of the asymmetries between legs obtained in the different tests carried out underscores the highly independent nature of the asymmetries according to the different tests involved, showing great variability of both inter-subject and intra-subject asymmetries for each test (Figure S1). Different studies would confirm the existence of great variability [8,11,39] and affirm that asymmetry is highly specific to the task—causing data analysis to be rather complex and making it difficult to determine a relationship with athlete performance. Moreover, the different evaluation methods, calculations of asymmetries and samples of athletes involved may explain the lack of consensus found in the scientific literature on asymmetries between legs and their association to athletic performance [13,39].

To date, most of the literature relating asymmetries to physical performance measures has made use of jumping tests to quantify the asymmetry component [7,40,43,44,45]; however, the magnitude of the asymmetry would also be highly dependent upon the executed jump type. For example, for horizontal jumps (such as single, triple and crossover hop tests), an asymmetry of 6–7% has been reported [43], which is consistent with the asymmetry of 7% shown by our athletes. In contrast, when asymmetry was evaluated through vertical jumps, a significant increase in asymmetry values was observed. Specifically, the asymmetries in single-leg countermovement jump showed asymmetry values of 10% [40,45], while evaluation by drop jump showed our athletes to yield asymmetry values of 13%, 15% and 18% for peak height, RSI and stiffness, respectively—with such values possibly reaching 50% in some athletes, in accordance with previous research [46]. Since the variables analyzed from the drop jump were those that showed a greater magnitude asymmetry, and considering our established threshold of 10% for defining an asymmetrical athlete, those variables were the ones that resulted in more athletes classified as being asymmetrical, with a total of 60% of our participants. Because of the reliance on both neurological and muscular mechanisms during DJ executions, a good RSI score is achieved through maximizing jump height and minimizing ground-contact time; hence, the RSI asymmetry will represent imbalances at the neuromuscular level between a player’s dominant and non-dominant side. In addition, it is evident that the magnitude of asymmetry is highly specific to the test performed, and this may be due to the absolute value obtained in each test and the equation used to calculate the asymmetry, since it has been observed that the tests in which a greater absolute result is achieved, fewer asymmetries are shown and vice versa. Therefore, if we want to define an asymmetry threshold for each player, it cannot be universal; it must be specific to each test. Furthermore, it should be noted that previous research [47] indicates that jump test values expressed as distance are not sensitive enough to detect meaningful limb differences.

In relation to functional asymmetries, our data evidenced a negative correlation between asymmetry in ankle dorsiflexion and anterior direction in YBT, which may be due because this range direction has a higher demand for mobility in ankle dorsiflexion [37]. Asymmetry in the mobility of the ankle has been previously related to different types of injuries such as damage to the anterior cruciate ligament (ACL) [48], also showing a strong association to the ability to accelerate and COD [17]—thereby showing that large asymmetry of such mobility may affect performance in the anterior reach direction of YBT. Interestingly, we observed that this same asymmetry in anterior YBT was that showing the closest relationship with the rest of the performance variables. In line with our results, previous research [11,49] would support that both the different ranges and the YBT composite are negatively associated with sports performance variables through jump height during a CMJ in basketball athletes, being relevant for this sport. Our study would add that the anterior YBT not only showed a negative correlation to the vertical jump variables but also correlated to jumps in the horizontal vector. Conversely, the posterior reach and YBT composite only showed a negative correlation to horizontal jump, which could be due to a similar movement pattern between posterior YBT and unilateral horizontal jump, and the greater demand of the posterior musculature in both the posterior directions of the YBT and the horizontal jumps [50]. In addition, high demands upon the posterior musculature are necessary for other actions decisive to the performance of these sports, such as COD or penetrations [2,3,50].

This study incorporates very interesting information, provides new evidence that has already been demonstrated, also including team sports such as handball, which have been little studied on this topic and are compared with other team sports, but despite the usefulness of our findings, the present study has several limitations. The relationship observed between the analyzed sports performances was weak or even disappeared when analyzed according to asymmetries. This could be due to the fact that, on one hand, a small sample was used and, on the other hand, the percentage of individuals with asymmetries was relatively small. In addition, the observed variability of asymmetry was very high even within the same sport and even within the same player, depending on the test performed [40]. Therefore, we recommend the use of a larger sample with asymmetry, in addition to analyzing such asymmetries by specific positions in order to obtain a greater homogeneity of results so that clearer conclusions can be obtained. Finally, it should be added that there is no clear consensus on how to measure asymmetries, given the variety of implemented equations and the different magnitudes obtained [17,34]—which makes it difficult to draw firm conclusions on the topic.

## 5. Conclusions

To sum up, the present study shows that athletes of similar team sports presented different levels of both strength in the vertical and horizontal jump, and dynamic balance and mobility analyzed, though this did not occur for the asymmetry between legs, it is important for the coach or physical trainer to consider these findings. The asymmetries showed important dispersion that would not depend on the type of sport involved but on each individual and on the applied test. On the other hand, although the asymmetries showed important variability, our results indicate that large asymmetries, mainly in the previous anterior direction of YBT, might negatively affect athletic performance through the vertical and horizontal jump. Trainers and physical trainers, therefore, could consider the possibility of intervening with the aim of reducing the identified asymmetries. From a training perspective, previous literature has suggested that unilateral training may afford great improvements in asymmetry between legs [1].

## Figures and Tables

**Table 1:** Sample data description.

Variables	Mean	SD	CL 95% (Lower; Upper)		Range
Age (years)	18.9	4.11	17.5	20.2	16–32
Weight (kg)	78.9	13.0	74.6	83.2	52–112
Height (m)	1.82	0.07	1.79	1.85	1.6–2.0
BMI (kg·m2–1)	23.7	3.15	22.6	24.6	16–29
Fat mass (%)	12.9	6.80	10.7	15.2	3.6–27
**Mobility**					
WB-DF R (°)	40.0	5.59	38.2	41.8	31–54
WB-DF L (°)	39.7	5.65	37.8	41.5	27–51
WB-DF C (°)	39.8	5.34	38.1	41.6	29–52
**Dynamic balance**					
YBT A R (cm)	51.2	8.26	48.5	53.9	33–68
YBT A L (cm)	51.9	7.70	49.3	54.4	34–68
YBT PM R (cm)	69.8	9.80	66.6	73.0	49–89
YBT PM L (cm)	68.0	7.88	65.4	70.6	50–85
YBT PL R (cm)	68.2	11.3	64.4	71.9	34–80
YBT PL L (cm)	69.5	11.1	65.8	73.1	49–89
YBT R (cm)	63.0	8.54	60.2	65.8	45–77
YBT L (cm)	63.1	8.10	60.4	65.7	47–79
**Lower limb strength**					
CMJ (cm)	35.9	5.19	34.2	37.7	26–45
DJU R (cm)	18.1	3.55	16.9	19.3	11–27
DJU L (cm)	18.3	4.27	16.8	19.6	9.8–29
RSI R	0.88	0.23	0.80	0.95	0.4–1.4
RSI L	0.86	0.24	0.78	0.94	0.5–1.5
Stiffness R	7.35	3.38	6.24	8.46	2.2–15.5
Stiffness L	7.12	2.97	6.14	8.09	2.4–14.1
THT R (cm)	578.8	57.7	559.8	597.8	457–738
THT L (cm)	593.9	67.3	571.8	616.0	467–799

Abbreviations: kg—kilograms; m—meter; BMI—body mass index; WB-DF—weight-bearing dorsiflexion; R—right; L—left; C—composite; cm—centimeters; YBT A—Y-balance test anterior reach; YBT PM—Y-balance test posteromedial reach; YBT PL—Y-balance test posterolateral reach; YBT—Y-balance test composite of all directions; CMJ—countermovement jump DJU—drop jump unilateral; RSI—reactive strength index; THTU—triple hop test unilateral; SD—standard deviation; CL—confidence limit.

**Table 2:** Comparative analysis of mobility, dynamic balance and lower limb strength according to the type of sport.

Variables	BB (n = 23)		HB (n = 25)		% (CL 90%)	ES (CL 90%)	Chances (%)	Outcome
	Mean	SD	Mean	SD				
**Mobility**								
WB-DF R (°)	39.0	5.27	41.5	5.68	6. 36 (−0.21; 13.4)	0.46 (−0.02; 0.95)	1/17/82	Likely
WB-DF L (°)	39.3	4.78	40.6	6.16	2.96 (−3.88; 10.4)	0.23 (−0.31; 0.76)	9/37/53	Unclear
WB-DF C (°)	39.1	4.70	41.1	5.72	4.62 (−1.71; 11.4)	0.37 (−0.14; 0.89)	3/25/71	Possibly
**Dynamic balance**								
YBT A R (cm)	53.7	6.5	47.9	7.5	−11.3 (−17.2; −4.91)	−0.93 (−1.47; −0.39)	99/1/0	Very likely
YBT A L (cm)	54.4	7.2	47.1	6.4	−13.3 (−18.8; −7.41)	−1.02 (−1.49; −0.55)	100/0/0	Most likely
YBT PM R (cm)	72.1	9.85	65.0	9.33	−9.85 (−16.1; −3.11)	−0.69 (−1.18; −0.21)	95/4/0	Very likely
YBT PM L (cm)	69.0	8.89	64.4	5.96	−6.23 (−11.3; −0.86)	−0.47 (−0.88; −0.06)	87/13/0	Likely
YBT PL R (cm)	71.6	9.6	61.8	11.6	−14.6 (−21.8; −6.82)	−1.09 (−1.69; −0.49)	99/1/0	Very likely
YBT PL L (cm)	72.3	10.9	63.7	10.2	−12.0 (−18.8; −4.67)	−0.79 (−1.29; −0.30)	97/2/0	Very likely
YBT R (cm)	65.8	7.26	58.3	8.14	−11.8 (−17.2; −6.10)	−1.08 (−1.61; −0.54)	100/0/0	Most likely
YBT L (cm)	65.2	8.14	58.44	6.65	−10.2 (−15.4; −4.75)	−0.81 (−1.26; −0.37)	99/1/0	Very likely
**Lower limb strength**								
CMJ (cm)	35.2	4.87	36.8	5.71	4.25 (−3.45; 12.6)	0.28 (−0.24; 0.80)	6/33/60	Unclear
DJU R (cm)	17.5	2.51	18.5	4.13	4.13 (−5.41; 14.6)	0.27 (−0.38; 0.92)	11/31/57	Unclear
DJU L (cm)	16.7	3.37	19.9	4.13	19.4 (7.87; 32.1)	0.81 (0.35; 1.28)	0/2/98	Very likely
RSI R	0.96	0.25	0.73	0.17	−22.5 (−31.6; −12.1)	−0.91 (−1.36; −0.46)	99/1/0	Very likely
RSI L	0.90	0.28	0.78	0.16	−11.8 (−22.4; 0.19)	−0.40 (−0.81; 0.01)	79/20/1	Likely
Stiffness R	8.64	3.55	5.08	1.81	−39.5 (−51.1; −25.3)	−1.05 (−1.49; −0.60)	100/0/0	Most likely
Stiffness L	8.07	3.15	5.52	2.38	−32.1 (−45.1; −16.0)	−0.88 (−1.36; −0.40)	99/1/0	Very likely
THT R (cm)	566.2	50.1	598.9	73.5	5.46 (−0.45; 11.7)	0.57 (−0.05; 1.18)	2/14/84	Likely
THT L (cm)	583.7	61.3	599.6	77.5	2.48 (−3.71; 9.07)	0.22 (−0.35; 0.80)	11/36/53	Unclear

Abbreviations: BB—basketball; HB—handball; WB-DF—weight-bearing dorsiflexion; R—right; L—left; C—composite; YBT A—Y-balance test anterior reach; YBT PM—Y-balance test posteromedial reach; YBT PL—Y-balance test posterolateral reach; YBT—Y-balance test composite of all directions; CMJ—countermovement jump DJU—drop jump unilateral; RSI—reactive strength index; THTU—triple hop test unilateral; SD—standard deviation; CL—confidence limit; ES—effect size.

## Data Availability

The data that support the findings of this study are available from the corresponding author upon reasonable request.

## Supplementary Materials

The following are available online at https://www.mdpi.com/1660-4601/18/4/1866/s1, Figure S1: Fold asymmetry expressed as a percentage of mobility, dynamic balance and lower limb strength asymmetries according to the type of sport. The model was adjusted for age. YBT A—Y-balance test anterior reach; YBT PM—Y-balance test posteromedial reach; YBT PL—Y-balance test posterolateral reach; YBT—Y-balance test composite of all directions; DJU—drop jump unilateral; RSI—reactive strength index; STF—stiffness; THT—triple hop test.

## References

[1] Gonzalo-Skok O., Tous-Fajardo J., Suarez-Arrones L., Arjol-Serrano J.L., Casajús J.A., Mendez-Villanueva A. (2017). Single-Leg Power Output and Between-Limbs Imbalances in Team-Sport Players: Unilateral Versus Bilateral Combined Resistance Training. Int. J. Sport. Physiol. Perform..

[2] Taylor J.B., Wright A.A., Dischiavi S.L., Townsend M.A., Marmon A.R. (2017). Activity Demands During Multi-Directional Team Sports: A Systematic Review. Sport. Med..

[3] Petway A.J., Freitas T.T., Calleja-González J., Leal D.M., Alcaraz P.E. (2020). Training load and match-play demands in basketball based on competition level: A systematic review. PLoS ONE.

[4] Kramer T.A., Sacko R.S., Pfeifer C.E., Gatens D.R., Goins J.M., Stodden D.F. (2019). The association between the funtional movement screen, Y-balance test, and physical performance test in male and female high school athletes. Int. J. Sport. Phys. Ther..

[5] Bishop C., Berney J., Lake J., Loturco I., Blagrove R., Turner A., Read P. (2019). Bilateral Deficit During Jumping Tasks. J. Strength Cond. Res..

[6] Sabido R., Hernández-Davó J.L., Botella J., Navarro A., Tous-Fajardo J. (2017). Effects of adding a weekly eccentric-overload training session on strength and athletic performance in team-handball players. Eur. J. Sport Sci..

[7] Maloney S.J., Richards J., Nixon D.G.D., Harvey L.J., Fletcher I.M. (2017). Do stiffness and asymmetries predict change of direction performance?. J. Sport. Sci..

[8] Raya-González J., Bishop C., Gómez-Piqueras P., Veiga S., Viejo-Romero D., Navandar A. (2020). Strength, Jumping, and Change of Direction Speed Asymmetries Are Not Associated With Athletic Performance in Elite Academy Soccer Players. Front. Psychol..

[9] Keeley D.W., Plummer H.A., Oliver G.D. (2011). Predicting asymmetrical lower extremity strength deficits in college-aged men and women using common horizontal and vertical power field tests: A possible screening mechanism. J. Strength Cond. Res..

[10] Hart N.H., Nimphius S., Spiteri T., Newton R.U. (2014). Leg strength and lean mass symmetry influences kicking performance in Australian football. J. Sport. Sci. Med..

[11] Bishop C., Turner A., Read P. (2018). Effects of inter-limb asymmetries on physical and sports performance: A systematic review. J. Sport. Sci..

[12] Kyritsis P., Bahr R., Landreau P., Miladi R., Witvrouw E. (2016). Likelihood of ACL graft rupture: Not meeting six clinical discharge criteria before return to sport is associated with a four times greater risk of rupture. Br. J. Sport. Med..

[13] Maloney S.J. (2019). The Relationship Between Asymmetry and Athletic Performance: A Critical Review. J. Strength Cond. Res..

[14] Madruga-Parera M., Bishop C., Read P., Lake J., Brazier J., Romero-Rodriguez D. (2020). Jumping-based Asymmetries are Negatively Associated with Jump, Change of Direction, and Repeated Sprint Performance, but not Linear Speed, in Adolescent Handball Athletes. J. Hum. Kinet..

[15] Fort-Vanmeerhaeghe A., Montalvo A.M., Sitjà-Rabert M., Kiefer A.W., Myer G.D. (2015). Neuromuscular asymmetries in the lower limbs of elite female youth basketball players and the application of the skillful limb model of comparison. Phys. Ther. Sport.

[16] Exell T., Irwin G., Gittoes M., Kerwin D. (2017). Strength and performance asymmetry during maximal velocity sprint running. Scand. J. Med. Sci. Sport..

[17] Gonzalo-Skok O., Serna J., Rhea M.R., Marín P.J. (2015). Relationships between functional movement tests and performance tests in young elite male basketball players. Int. J. Sport. Phys. Ther..

[18] Barrera-Domínguez F.J., Almagro B.J., Tornero-Quiñones I., Sáez-Padilla J., Sierra-Robles Á., Molina-López J. (2020). Decisive Factors for a Greater Performance in the Change of Direction and Its Angulation in Male Basketball Players. Int. J. Environ. Res. Public Health.

[19] Balsalobre-Fernández C., Romero-Franco N., Jiménez-Reyes P. (2019). Concurrent validity and reliability of an iPhone app for the measurement of ankle dorsiflexion and inter-limb asymmetries. J. Sport. Sci..

[20] Onofrei R.R., Amaricai E., Petroman R., Suciu O. (2019). Relative and absolute within-session reliability of the modified Star Excursion Balance Test in healthy elite athletes. PeerJ.

[21] Bolgla L.A., Keskula D.R. (1997). Reliability of lower extremity functional performance tests. J. Orthop. Sport. Phys. Ther..

[22] De Blas X., Padullés J.M., Del Amo J.L.L., Guerra-Balic M. (2012). Creación y validación de Chronojump-Boscosystem: Un instrumento libre para la medición de saltos verticales. RICYDE Rev. Int. Cienc. Deport..

[23] Morin J.B., Dalleau G., Kyröläinen H., Jeannin T., Belli A. (2005). A simple method for measuring stiffness during running. J. Appl. Biomech..

[24] Cohen J. (1988). Statistical Power Analysis for the Behavioral Sciences: Jacob Cohen.

[25] Chtourou H., Hammouda O., Souissi H., Chamari K., Chaouachi A., Souissi N. (2012). Diurnal variations in physical performances related to football in young soccer players. Asian J. Sport. Med..

[26] Webb P., Lander J.L. (1983). An economical fitness testing battery for high school and college rugby teams. Sport. Coach.

[27] Hopkins W.G., Marshall S.W., Batterham A.M., Hanin J. (2009). Progressive Statistics for Studies in Sports Medicine and Exercise Science. Med. Sci. Sport. Exerc..

[28] Hopkins W.G. (2006). Spreadsheets for analysis of controlled trials, with adjustment for a subject characteristic. Sportscience.

[29] Dello Iacono A., Martone D., Milic M., Padulo J. (2017). Vertical- vs. Horizontal-Oriented Drop Jump Training. J. Strength Cond. Res..

[30] Abdelkrim N.B., El Fazaa S., El Ati J. (2007). Time-motion analysis and physiological data of elite under-19-year-old basketball players during competition. Br. J. Sport. Med..

[31] Michalsik L.B., Laver L., Landreau P., Seil R., Popovic N. (2018). On-Court physical demands and physiological aspects in elite team handball. Handball Sports Medicine: Basic Science, Injury Management and Return to Sport.

[32] Stojanović E., Stojiljković N., Scanlan A.T., Dalbo V.J., Berkelmans D.M., Milanović Z. (2018). The Activity Demands and Physiological Responses Encountered During Basketball Match-Play: A Systematic Review. Sport. Med..

[33] Dai B., Layer J., Vertz C., Hinshaw T., Cook R., Li Y., Sha Z. (2019). Baseline Assessments of Strength and Balance Performance and Bilateral Asymmetries in Collegiate Athletes. J. Strength Cond. Res..

[34] Bishop C., Turner A., Maloney S., Lake J., Loturco I., Bromley T., Read P. (2019). Drop Jump Asymmetry is Associated with Reduced Sprint and Change-of-Direction Speed Performance in Adult Female Soccer Players. Sports.

[35] Kozinc Z., Marković G., Hadžić V., Šarabon N. (2020). Relationship between force-velocity-power profiles and inter-limb asymmetries obtained during unilateral vertical jumping and singe-joint isokinetic tasks. J. Sport. Sci..

[36] Pardos-Mainer E., Casajús J.A., Gonzalo-Skok O. (2019). Adolescent female soccer players’ soccer-specific warm-up effects on performance and inter-limb asymmetries. Biol. Sport.

[37] Xixirry M.G., Riberto M., Manoel L.S. (2019). Analysis of y balance test and dorsiflexion lunge test in professional and amateur soccer players. Rev. Bras. Med. Esporte.

[38] Read P.J., Oliver J.L., De Ste Croix M.B.A., Myer G.D., Lloyd R.S. (2018). A prospective investigation to evaluate risk factors for lower extremity injury risk in male youth soccer players. Scand. J. Med. Sci. Sport..

[39] Fort-Vanmeerhaeghe A., Bishop C., Busca B., Aguilera-Castells J., Vicens-Bordas J., Gonzalo-Skok O. (2020). Inter-limb asymmetries are associated with decrements in physical performance in youth elite team sports athletes. PLoS ONE.

[40] Bishop C., Lake J., Loturco I., Papadopoulos K., Turner A., Read P. (2018). Interlimb Asymmetries: The Need for an Individual Approach to Data Analysis. J. Strength Cond. Res..

[41] Exell T.A., Irwin G., Gittoes M.J.R., Kerwin D.G. (2012). Implications of intra-limb variability on asymmetry analyses. J. Sport. Sci..

[42] Bishop C. (2020). Interlimb Asymmetries: Are Thresholds a Usable Concept?. Strength Cond. J..

[43] Dos’Santos T., Thomas C., Jones P.A., Comfort P. (2017). Asymmetries in single and triple hop are not detrimental to change of direction speed. J. Trainology.

[44] Hoffman J.R., Ratamess N.A., Klatt M., Faigenbaum A.D., Kang J. (2007). Do bilateral power deficits influence direction-specific movement patterns?. Res. Sport. Med..

[45] Lockie R.G., Callaghan S.J., Berry S.P., Cooke E.R.A., Jordan C.A., Luczo T.M., Jeffriess M.D. (2014). Relationship between unilateral jumping ability and asymmetry on multidirectional speed in team-sport athletes. J. Strength Cond. Res..

[46] Maloney S.J., Fletcher I.M., Richards J. (2016). A comparison of methods to determine bilateral asymmetries in vertical leg stiffness. J. Sport. Sci..

[47] Kotsifaki A., Korakakis V., Whiteley R., Van Rossom S., Jonkers I. (2020). Measuring only hop distance during single leg hop testing is insufficient to detect deficits in knee function after ACL reconstruction: A systematic review and meta-analysis. Br. J. Sport. Med..

[48] Wahlstedt C., Rasmussen-Barr E. (2015). Anterior cruciate ligament injury and ankle dorsiflexion. Knee Surgery Sport. Traumatol. Arthrosc..

[49] Read P.J., Hughes J., Stewart P., Chavda S., Bishop C., Edwards M., Turner A.N. (2014). A needs analysis and field-based testing battery for basketball. Strength Cond. J..

[50] Nygaard Falch H., Guldteig Rædergård H., Van den Tillaar R. (2020). Relationship of Performance Measures and Muscle Activity between a 180° Change of Direction Task and Different Countermovement Jumps. Sports.

