# Supplementary material for: Influence of Dynamic Balance on Jumping-Based Asymmetries in Team Sport: A between-Sports Comparison in Basketball and Handball Athletes
Source: Int J Environ Res Public Health. 2021 Feb 14;18(4):1866. doi: 10.3390/ijerph18041866 (PMC7917681; doi:10.3390/ijerph18041866)
Supplement: Supplementary file 1 [file ijerph-18-01866-s001.pdf]

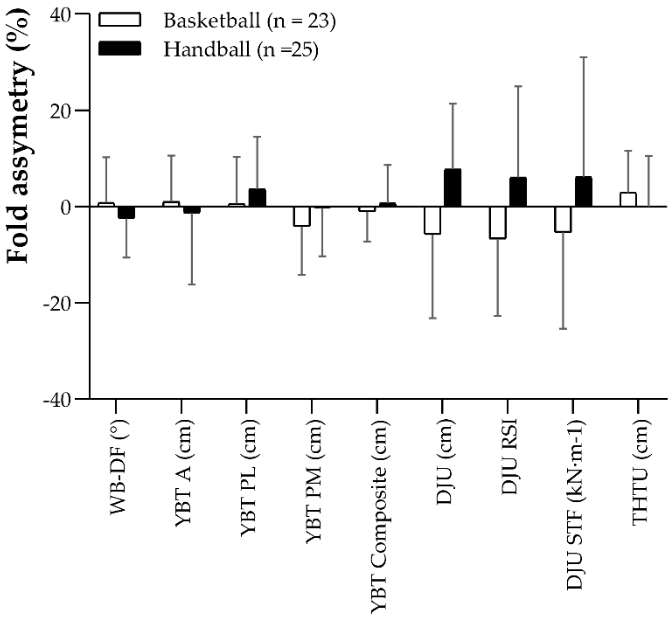

Figure S1: Fold asymmetry expressed as percentage of mobility, dynamic balance and lower limb strength asymmetries according to the type of sport. The model has been adjusted for age. WB-DF—weight-bearing dorsiflexion; YBT A—Y-balance test anterior reach; YBT PM—Y-balance test posteromedial reach; YBT PL—Y-balance test posterolateral reach; YBT—Y-balance test composite of all directions; DJU—drop jump unilateral; RSI—reactive strength index; THTU—triple hop test unilateral.
